# Plant-Mediated Effects on Mosquito Capacity to Transmit Human Malaria

**DOI:** 10.1371/journal.ppat.1005773

**Published:** 2016-08-04

**Authors:** Domonbabele F. d. S. Hien, Kounbobr R. Dabiré, Benjamin Roche, Abdoulaye Diabaté, Rakiswende S. Yerbanga, Anna Cohuet, Bienvenue K. Yameogo, Louis-Clément Gouagna, Richard J. Hopkins, Georges A. Ouedraogo, Frédéric Simard, Jean-Bosco Ouedraogo, Rickard Ignell, Thierry Lefevre

**Affiliations:** 1 Institut de Recherche en Sciences de la Santé (IRSS), Bobo Dioulasso, Burkina Faso; 2 UMISCO lab (Unité de Modélisation Mathématique et Informatique des Systèmes Complexes), UMI IRD/UPMC 209, Bondy, France; 3 MIVEGEC lab (Maladies Infectieuses et Vecteurs: Ecologie, Génétique, Evolution et Contrôle), UMR Université Montpellier, CNRS 5290, IRD 224, 911 Av. Agropolis, Montpellier, France; 4 University of Greenwich, Natural Resource Institute–Department of Agriculture Health and Environment, Chatham Maritime, Kent, United Kingdom; 5 Université Polytechnique de Bobo Dioulasso, Bobo Dioulasso, Burkina Faso; 6 Unit of Chemical Ecology, Department of Plant Protection Biology, Swedish University of Agricultural Sciences, Alnarp, Sweden; Institut Pasteur, FRANCE

## Abstract

The ecological context in which mosquitoes and malaria parasites interact has received little attention, compared to the genetic and molecular aspects of malaria transmission. Plant nectar and fruits are important for the nutritional ecology of malaria vectors, but how the natural diversity of plant-derived sugar sources affects mosquito competence for malaria parasites is unclear. To test this, we infected *Anopheles coluzzi*, an important African malaria vector, with sympatric field isolates of *Plasmodium falciparum*, using direct membrane feeding assays. Through a series of experiments, we then examined the effects of sugar meals from *Thevetia neriifolia* and *Barleria lupilina* cuttings that included flowers, and fruit from *Lannea microcarpa* and *Mangifera indica* on parasite and mosquito traits that are key for determining the intensity of malaria transmission. We found that the source of plant sugar meal differentially affected infection prevalence and intensity, the development duration of the parasites, as well as the survival and fecundity of the vector. These effects are likely the result of complex interactions between toxic secondary metabolites and the nutritional quality of the plant sugar source, as well as of host resource availability and parasite growth. Using an epidemiological model, we show that plant sugar source can be a significant driver of malaria transmission dynamics, with some plant species exhibiting either transmission-reducing or -enhancing activities.

## Author Summary

The *Anopheles coluzzii* mosquito is an effective vector of human *Plasmodium falciparum* malaria. Besides feeding on blood, females readily feed on natural sources of plant sugars; but how plant diversity affects their ability to transmit malaria parasites is currently unknown. Here we show that mosquito feeding on peridomestic plant- and fruit-derived sugar sources can affect its interaction with *P*. *falciparum*, and hence malaria transmission. The planting of anti-*Plasmodium* plant sugar sources may represent a promising alternative strategy to contribute to the control of malaria.

## Introduction

The ability of anopheline mosquitoes to transmit *Plasmodium falciparum* malaria is a complex phenotypic trait, determined by mosquito and parasite genetic factors, environmental factors, as well as the interaction between these factors [1–6]. A key environmental variable for host-parasite relationships is the availability and quality of food resources, which have been shown to influence host susceptibility, parasite infectivity and virulence, and ultimately disease dynamics [7]. The influence of diet on infectious diseases is particularly apparent in tritrophic interactions involving herbivorous insects, their parasites and larval food plants [8]. Such plant-mediated effects have often been attributed to either the direct toxic effect of plant secondary metabolites on parasite development [9], or differences in nutritional value that, in turn, affect host immunocompetence [10].

Mosquitoes, like herbivorous insects, are part of a multitrophic system that includes plants and parasites. Although female mosquitoes are well known blood-feeders, sugar sources such as nectar from floral and extra-floral nectaries, fruits and phloem sap compose an important part of their diet and have significant biological implications [11,12]. Recent studies indicate that females of *An*. *gambiae s*.*l*., the main vector of *P*. *falciparum* in large areas of Africa, can locate and display preference for natural sources of plant sugar [13–16], and that environmental sugars may play a crucial role in malaria vectorial capacity (a standard measure of malaria transmission potential), through effects on mosquito survival [17–24] or blood-feeding rate [17,21–23]. However, feeding on plant tissues could also influence vectorial capacity by enhancing or mitigating infection in malaria mosquitoes.

Compared to the important efforts devoted to documenting plant-mediated interactions among herbivorous insects and their parasites [8], studies on the nutritional effects in mosquito-*Plasmodium* interactions have lagged behind despite obvious epidemiological importance. The few existing studies indicate that adult diet can indeed influence mosquito immunocompetence and susceptibility to malaria parasites [25–30]. However, these studies have focused on quantitative, as opposed to qualitative, changes in diet, and have used non-natural food sources (e.g. different concentrations of glucose solutions). Whether natural plant diversity affects mosquito susceptibility to malaria parasites therefore remains to be discovered.

The current study addresses questions of the impact of natural plant diversity on mosquito susceptibility to malaria parasites, using the natural tritrophic interaction between the parasite *P*. *falciparum*, responsible for causing the most severe form of human malaria, the mosquito *An*. *coluzzii* (formerly the M molecular form of *An*. *gambiae s*.*s*.), a major vector of *P*. *falciparum* in Africa, and a range of peridomestic plant- and fruit-derived sugar sources. We challenged *An*. *coluzzi* females with sympatric field isolates of *P*. *falciparum* using direct membrane feeding assays and, through a series of experiments, examined the effects of plant sugar sources on parasite and mosquito traits that are instrumental in determining the intensity of malaria transmission: (i) the early parasite development within mosquito guts, (ii) the proportion of mosquitoes harboring sporozoite transmissible stages (i.e. the sporozoite index), (iii) the extrinsic incubation period of the parasite (EIP), (iv) the survival and fecundity of infected mosquitoes, and (v) the costs and benefits to feed on these plant sugar sources for both infected and uninfected mosquitoes. Finally, these results were combined into an epidemiological model to predict the relative contribution of different plant species to overall malaria transmission. We provide evidence for malaria transmission-reducing and -enhancing activities of some natural plant sugar sources.

## Methods

### Mosquitoes

Laboratory-reared *An*. *coluzzii* were obtained from an outbred colony established in 2008 and repeatedly replenished with F1 from wild-caught mosquito females collected in Kou Valley (11°23'14"N, 4°24'42"W), 30 km from Bobo Dioulasso, south-western Burkina Faso (West Africa), and identified by routine PCR-RFLP. Mosquitoes were held in 30 cm × 30 cm × 30 cm mesh-covered cages at the IRSS insectary under standard conditions (27 ± 2°C, 70 ± 5% relative humidity, 12:12 LD). Females were maintained on rabbit blood by direct feeding, and adult males and females fed with 5% glucose. Larvae were reared at a density of about 300 first instar larvae in 700 ml of water in plastic trays and were fed with Tetramin Baby Fish Food (Tetrawerke, Melle, Germany).

### Plant materials and mosquito feeding on plant sugar source

We selected two perennial flowering ornamental plants: *Thevetia neriifolia* and *Barleria lupilina* collected in the gardens and parkland of Bobo Dioulasso (S1A and S1B Fig); and two fruits: mango (*Mangifera indica*, variety “demoiselle”) and *Lannea microcarpa*, both locally produced and purchased from the market in Bobo Dioulasso (S1C and S1D Fig). The plants and fruits were selected based on (i) their wide distribution around human dwellings in villages and cities of western Burkina Faso, (ii) *An*. *coluzzii* females readily rest, probe and feed on them (inferred from both field and lab observations, S1 appendix and [31]), and (iii) they provide relatively high mosquito survival to allow for the development of *Plasmodium falciparum*.

Plant sugar sources were offered to mosquitoes in 30 cm × 30 cm × 30 cm mesh-covered cages. For *T*. *neriifolia* and *B*. *lupilina*, between five and ten fresh bundles of flowering cuttings were added within each cage. The base of the bunch was wrapped in moistened paper towels and an aluminum sheet (S2A and S2B Fig). For the mango treatment, ripe fruits were cut in half, held on two 20 cm long wooden stakes and individually placed in the mosquito cages (S2C Fig). One bunch of about 40 ripe *L*. *microcarpa* fruits was placed in a petri dish within the mosquito cages (S2D Fig). Finally, 5% glucose was provided on cotton pads wrapped around two 20 cm long wooden stick (S2E Fig). Cuttings, fruits, and glucose were replaced every day. To confirm sugar ingestion, the gut content of a subset of mosquitoes was determined by the cold anthrone test for fructose [32] (S1 Appendix). Mosquitoes can sugar feed from a wide range of plant parts (floral nectaries, extra-floral nectaries at the base of the flowers or on stems or leaves, tissues juices, phloem sap, and honeydew) [11]. Here, the exact sources of the sugar taken up by mosquito females on plant cuttings (*T*. *neriifolia* and *B*. *lupilina*) were not determined.

### Parasite isolate and experimental infections

*Plasmodium falciparum* gametocyte carriers were recruited among 5–12-year-old school children in the villages of Soumousso and Dande, located respectively 30 km north east and 60 km North West of Bobo Dioulasso in southwestern Burkina Faso. Parasitological surveys were carried out in collaboration with the medical team in charge of malaria treatment at the local health center in these two villages. Thick blood smears were taken from each volunteer, air-dried, Giemsa-stained, and examined by microscopy for the presence of *P*. *falciparum* at the IRSS lab in Bobo Dioulasso. Asexual trophozoite parasite stages were counted against 200 leucocytes, while infectious gametocytes stages were counted against 1000 leukocytes. Children with asexual parasitemia of > 1,000 parasites per microliter (estimated based on an average of 8000 leucocytes/ml) were treated in accordance with national guidelines. Asymptomatic *P*. *falciparum* gametocyte-positive children were recruited for the study. Mosquito infections were performed by direct feeding membrane assays using whole donor blood (i.e. no serum replacement) [33,34]. Briefly, gametocyte carrier blood was collected by venipuncture into heparinized tubes. Three to four day old female mosquitoes, distributed in 500 ml paper cups at a density of 80 mosquitoes per cup, were allowed to feed on this blood for one hour. Mosquitoes were starved of glucose solution for 24 h prior to the infection, with only water available, in wet cotton wool. Non-fed or partially fed females were removed and discarded, while the remaining fully-engorged mosquitoes were maintained in a biosafety room under the same standard conditions of 27 ± 2°C, 70 ± 5% relative humidity and, 12:12 LD) on their assigned plant sugar source.

### Experiment 1: To determine the effect of plant sugars on the early development of *P*. *falciparum* and on mosquito survival and fecundity

Upon emergence, batches of female mosquitoes from the colony were randomly assigned to one of four sugar sources: a 5% glucose solution, *T*. *neriifolia*, *B*. *lupilina* or *M*. *indica*, for three to four days. Thus, mosquitoes were given ample time to feed on their sugar source, and by 2–3 days, all mosquitoes would have acquired sugar meal at least once. Male and female mosquitoes were kept together to ensure insemination. Three to four day old females were then transferred to paper cups for infection (see above). After infection, fully-engorged females were placed in 30 cm × 30 cm × 30 cm mesh-covered cages with their assigned sugar source (S2 Fig). Females thus received the sugar treatment both before and after the infection. Seven days post blood-meal, mosquitoes were dissected to assess microscopically (×400) the presence of oocysts in the midgut stained with 2% mercurochrome. We also examined whether ovaries contained matured eggs (egg incidence). Mosquito survival was monitored from 1 to 7 days post-treatment (dpi). Twice a day, dead mosquitoes were removed and counted from each cage at 08:00 and 17:00. We performed four replicates using a total of seven gametocyte carriers. To avoid potential cage effect on survival, fecundity and susceptibility to infection, mosquitoes belonging to the same sugar treatment and fed on blood from the same gametocyte carrier were distributed in at least two different cages. On average, 30 (range 10–50) mosquitoes per sugar treatment and gametocyte carrier were dissected (except for the mango treatment, gametocyte carriers A and B for which only 1 and 4 mosquitoes were dissected, respectively, due to a low survival rate, see details in S1 Table).

### Experiment 2: To determine the effect of plant sugars on sporozoite index and EIP

The same general procedure as that described in Experiment 1 was used in Experiment 2. Because *P*. *falciparum* takes an average of 14 days to disseminate sporozoite, and mosquito survival rate on *M*. *indica* was relatively low, we chose to replace this fruit with *L*. *microcarpa*. As in the previous experiment, mosquitoes were offered their assigned sugar sources both before and after the infection. Mosquitoes were sampled from day 9 to 13 dpi by dissecting the head and thorax of 20 mosquitoes from each treatment and time point to estimate the parasite’s extrinsic incubation period. On day 14 the remaining mosquitoes in each cage were similarly dissected. The head and thorax were individually stored at -20°C. Sporozoite dissemination in head and thorax was assessed using PCR [35]. Mosquito survival was monitored from 1 to 14 dpi. Twice a day, dead mosquitoes were removed and counted from each cage at 08:00 and 17:00. We performed 2 replicates using a total of 4 gametocyte carriers between April and June 2013. On average, 26 (range 8–50) mosquitoes per sugar treatment and gametocyte carrier were dissected (see details in S2 Table).

### Experiment 3: To determine how parasite infection and host plant species interact to influence mosquito longevity

The general procedure was identical to that of the two previous experiments except that a group of uninfected control mosquitoes was added, and that survival was monitored until all the mosquitoes had died. We assigned female mosquitoes to a 5% glucose solution, *M*. *indica*, *T*. *neriifolia* or *B*. *lupilina*. Uninfected control mosquitoes received heat-treated gametocytic blood. Briefly, half of the venous blood drawn from the gametocyte carrier was heated at 45°C for 20 minutes to kill the parasite gametocytes. Such heat treatment does not affect the survival and fecundity of mosquito females [36]. Because the nutritive quality of blood substantially vary among people and especially between infected and uninfected individuals, this procedure allows experiments to avoid the potential confounding effects of different blood origins on the performance of infected and control mosquitoes [33,37].

### Statistical analysis

All statistical analyses were performed in R (version 2.15.3). Logistic regression by Generalized Linear Mixed Models (GLMM, binomial errors, logit link; lme4 package) was used to investigate the effect of sugar treatment on oocyst infection rate (Experiment 1) and sporozoite index (Experiment 2). GLMM with negative binomial errors (glmmADMB package) was used to test the effect of sugar treatment on oocyst intensity (Experiment 1). For these GLMMs, full models included sugar treatment and gametocytemia and their interaction as fixed effects. Binomial GLMM was also used to test the effect of sugar treatment, infection and gametocytemia on mosquito egg incidence (presence/absence of mature eggs in ovaries, Experiment 1). In all GLMMs, gametocyte carrier identity was included as a random effect. The effect of sugar treatment on mosquito survivorship was analyzed using Cox’s proportional hazard regression models ("coxph" function in the "survival" package) with (experiments 1 and 2) or without (experiment 3) censoring. Sugar treatment, gametocytemia, infection (for experiment 3 only) and their interaction were considered as explanatory variables. Model simplification used stepwise removal of terms, followed by likelihood ratio tests (LRT). Term removals that significantly reduced explanatory power (*p* < 0.05) were retained in the minimal adequate model.

### Mathematical modeling

To quantify the consequences of plant sugar source on malaria transmission, we designed a mathematical model, grounded within the SIR framework [38,39], that accounts for the essential transmission processes: vector competence (i.e. infection level) and infectious potential (the period during which the mosquito can transmit the pathogen). We assumed that the mosquito population studied could be categorized into Susceptible individuals (S_m_, i.e. that could be infected), which then moved to the Exposed category upon infection (E_m_, i.e. infected, but not yet infectious) and became Infectious (I_m_, i.e. mosquitoes that can transmit the pathogen). Infectious potential was characterized by two phases: a first phase of low infection level (when the oocysts crack up and sporozoites begin to invade mosquito salivary glands i.e. day 9 to days 10–11) followed by a second phase of higher infection levels (from day 10–11 until mosquito death). The duration and vector competence for each phase and each plant are detailed in the S2 Appendix. We also assumed similar categories for the human population (S2 Appendix). We simulated the expected outbreak size in a human population (number of individuals that has been infected at the end of the season when one infectious human was introduced into a population of 100 individuals) for each plant sugar. We explored the parameter space through a Latin hypercube sampling with 10,000 replicates for which all parameters were randomly chosen within their confidence interval based on the data measurements obtained experimentally here. Data are deposited in the Dryad repository: (DOI: doi:10.5061/dryad.9s690) [40]

### Ethics statement

Ethical approval was obtained from the Centre Muraz Institutional Ethics Committee (A003-2012/CE-CM) and National Ethics Committee of Burkina Faso (2014–0040). Before enrollment, legal guardians of each child participant provided written consent on behalf of the minors. The protocol conforms to the declaration of Helsinki on ethical principles for medical research involving human subjects (version 2002).

This study was carried out in strict accordance with the recommendations in the Guide for the Care and Use of Laboratory Animals of the National Institutes of Health. The protocol was approved by both the Office of Laboratory Animal Welfare of US Public Health Service (Assurance Number: A5928-01) and national committee of Burkina Faso (IRB registration #00004738 and FWA 00007038). Animals were cared for by trained personnel and veterinarians.

## Results

### Plant sugar source influences the early development of *P*. *falciparum* and the vector’s survival and fecundity

**Fig 1:**
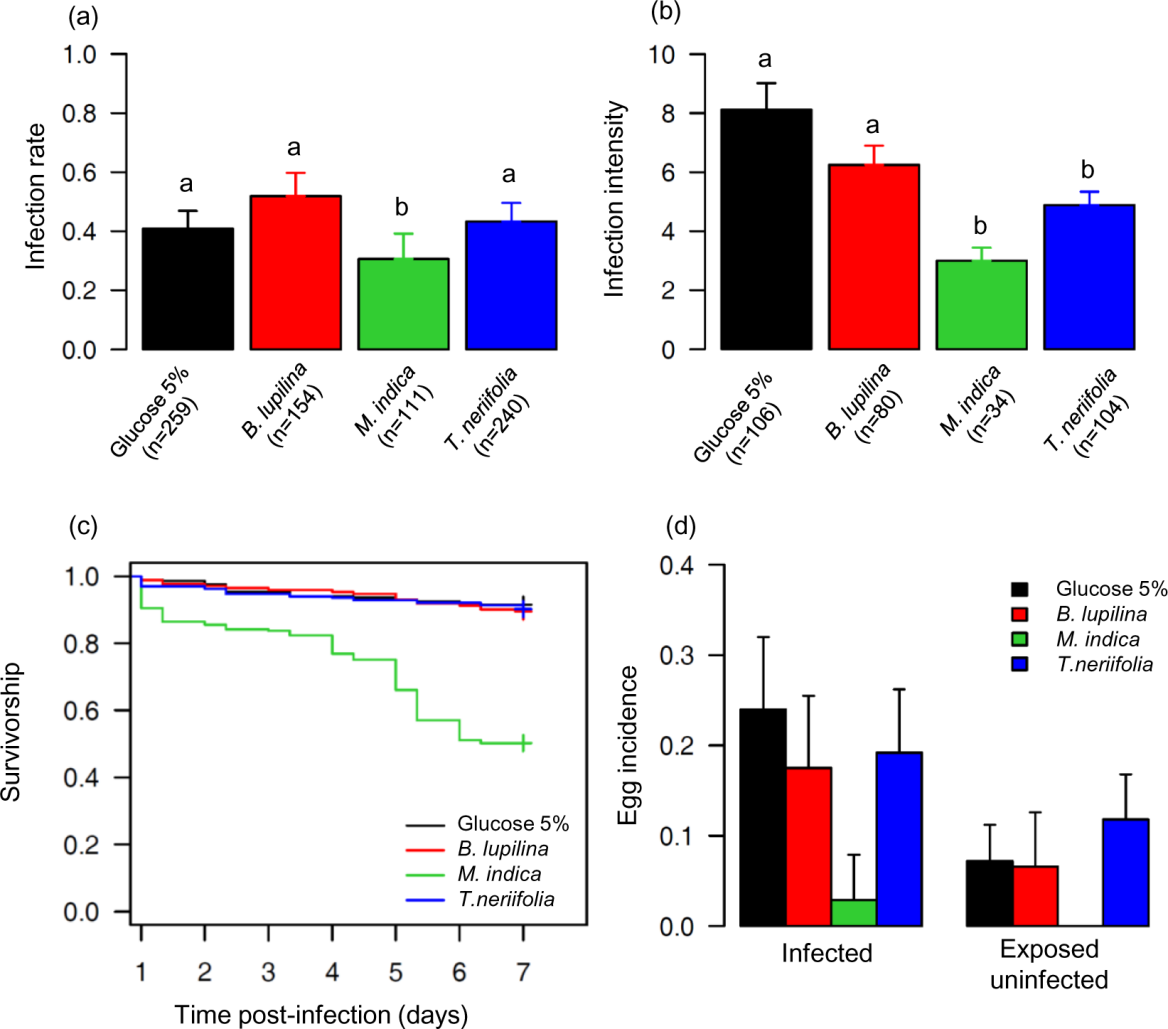
Effect of sugar treatment on the early development of *P*. *falciparum*, and on the survival and fecundity of malaria-exposed *Anopheles coluzzii*. (a) Infection rate (± 95% CI), expressed as the proportion of mosquitoes exposed to an infectious blood meal and harboring at least one oocyst in their midgut, over 4 replicates and using a total of 7 gametocyte carriers. Numbers in brackets indicate the total number of mosquitoes dissected 7 days post infection (dpi) for each sugar treatment. Different letters above the bars denote statistically significant differences based on multiple pair-wise post-hoc tests. (b) Infection intensity (± se), expressed as the mean number of developing oocysts in the guts of infected females, over 4 replicates and using a total of 7 gametocyte carriers. Numbers in brackets indicate the total number of infected mosquitoes for each sugar treatment. Different letters above the bars denote statistically significant differences based on multiple pair-wise post-hoc tests. (c) Survivorship of malaria-exposed mosquitoes for each sugar treatment over 4 replicates and using a total of 7 gametocyte carriers. Survival was recorded twice a day from 1 to 7 dpi. (d) Egg incidence (± 95% CI) of malaria-exposed mosquitoes, expressed as the proportion of mosquito females carrying fully matured eggs inside their ovaries on 7 dpi for each sugar treatment and infection status.

A total of 324 out of 764 (42.4%) *An*. *coluzzii* became successfully infected upon parasite exposure. The infection rate significantly varied among mosquitoes maintained on different sugar treatments (LRT *X* ^*2*^_3_ = 13.2, P = 0.004; Fig 1A). Infection dose (i.e. gametocytemia) significantly affected parasite prevalence, with higher gametocyte density in blood leading to an increased likelihood of infection (*X* ^*2*^_1_ *inline* = 5.2, P = 0.04). Finally, there was a significant gametocytemia by sugar treatment interaction (*X* ^*2*^_3_ *inline* = 10.2, P = 0.017).

The mean number of developing oocysts in infected females (i.e. intensity) significantly varied among sugar treatments (LRT *X* ^*2*^_3_ = 19.8, P = 0.0002, Fig 1B). Gametocytemia had a positive effect on intensity (LRT *X* ^*2*^_1_ = 4.6, P = 0.03), and there was a significant gametocytemia by sugar treatment interaction (LRT *X* ^*2*^_3_ = 12.5, P = 0.006).

Sugar source was also linked to the probability of a mosquito surviving until 7 dpi (LRT *X* ^*2*^_3_ = 80, P < 0.001; Fig 1C and S3 Table), with survivorship ranging from 92%, 90% and 89.5% on 5% glucose, *T*. *neriifolia* and *B*. *lupilina*, respectively, to 50% on *M*. *indica*. Egg incidence was reduced in mosquitoes fed on *M*. *indica* (LRT *X* ^*2*^_3_ = 27, P < 0.001; Fig 1D and S3 Table). Infected females were more likely to carry eggs in their ovaries compared to females exposed to an infectious blood-meal but which remained uninfected (hereafter referred to as “exposed-uninfected”) (LRT *X* ^*2*^_1_ = 8, P = 0.005; Fig 1D), irrespective of sugar treatment (i.e. no infection by sugar treatment interaction: LRT *X* ^*2*^_3_ = 3.5, P = 0.32, Fig 1D). Among infected females, egg incidence was also positively associated with infection intensity (X^2^_1_ = 5.8, P = 0.016, S3 Fig). Finally, there was no effect of gametocytemia on mosquito survival (LRT *X* ^*2*^_1_ = 0.12, P = 0.72) and egg incidence (LRT *X* ^*2*^_1_ = 1.3, P = 0.26).

### Plant sugar source influences the sporozoite index and the parasite’s Extrinsic Incubation Period (EIP)

The aim of this experiment was to determine the extent to which natural plant sugar sources can affect the time of sporozoite release as well as the proportion of mosquitoes harboring sporozoites. A high mortality rate was observed in mosquitoes fed on *M*. *indica* (Fig 1C and preliminary data indicating a very low number of mosquitoes surviving until 14 dpi, the average incubation period of *P*. *falciparum*), hence, we replaced *M*. *indica* with *Lannea microcarpa* (S1 Fig), a less common fruit-derived sugar but one which provides relatively high mosquito survival rate.

**Fig 2:**
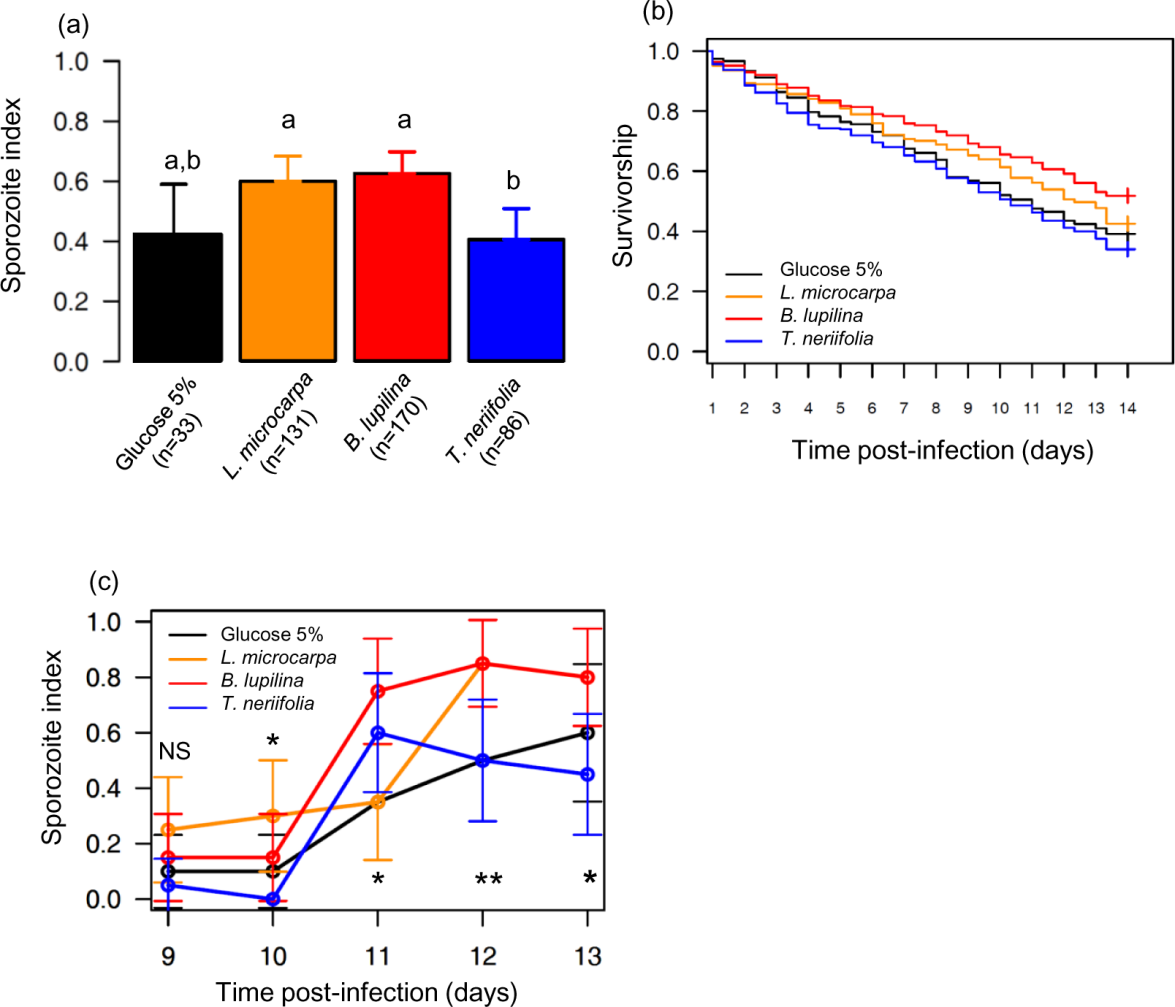
Effect of sugar treatment on the sporozoite index and EIP. (a) Sporozoite index (± 95% CI), expressed as the proportion of mosquitoes exposed to an infectious blood meal and having disseminated sporozoites in their head/thoraces, over 2 replicates and using a total of 4 gametocyte carriers. Numbers in brackets indicate, for each sugar treatment, the total number of mosquitoes analyzed with PCR on 14 days post infection (dpi). Different letters above the bars denote statistically significant differences based on multiple pair-wise post-hoc tests. (b) Survivorship of malaria-exposed mosquitoes for each sugar treatment over 2 replicates, and using a total of 4 gametocyte carriers. Survival was recorded twice a day from 1 to 14 dpi. (c) Sporozoite index (± 95% CI) over time and using a total of 2 gametocyte carriers. *p<0.05; **p < 0.01, NS: non-significant difference between sugar treatment

Overall, a total of 235 out of 420 (56%) *An*. *coluzzii* harbored sporozoites 14 dpi. Sugar treatment had a significant effect on sporozoite index (LRT *X* ^*2*^_3_ = 15; P = 0.002; Fig 2A), with mosquitoes fed on *T*. *neriifolia* being less likely to harbor disseminated sporozoites (Fig 2A). Gametocytemia positively influenced sporozoite index (LRT *X* ^*2*^_1_ = 4, P = 0.046). We also found an effect of sugar treatment on mosquito survival (LRT *X* ^*2*^_3_ = 26; P < 0.001, Fig 2B and S3 Table) with mosquitoes living longer when maintained on *B*. *lupilina* followed by *L*. *microcarpa*, 5% glucose and *T*. *neriifolia*.

Sporozoites were observed as soon as 9 dpi (Fig 2C). Significant differences in sporozoite index between sugar treatments were observed from 10 to 14 dpi, indicating that the temporal dynamics of sporozoite dissemination varied (Fig 2C) depending on sugar treatment. As expected, there was a significant time effect with sporozoite index increasing over time (LRT *X* ^*2*^_1_ = 94, P < 0.0001).

### Plant sugar source similarly influences the longevity of infected and uninfected control mosquitoes

The previous experiment showed a reduced sporozoite index in *T*. *neriifolia*-fed mosquitoes (Fig 2A) compared to those fed on other sugar sources. Although, this can be an effect of the plant on parasite development and sporozoite dissemination, this observation is also consistent with an increased mortality of infected individuals compared to exposed-uninfected counterparts. In other words, there might be an interaction between infection and plant treatment, such that infected mosquitoes suffer increased mortality when reared on this species, and hence display reduced sporozoite index at 14 dpi. Therefore, we measured mosquito longevity using a factorial design, crossing infection status (infected with *P*. *falciparum* versus exposed-uninfected versus uninfected control) with sugar treatment. Uninfected control mosquitoes received a heat-treated gametocytic blood meal. Because *M*. *indica* showed an effect on both oocyst infection rate and intensity (Fig 1A and 1C), while *L*. *microcarpa* showed no effect on sporozoite dissemination (Fig 2A) compared to a 5% glucose solution, we used *M*. *indica* instead of *L*. *microcarpa* for this experiment.

**Fig 3:**
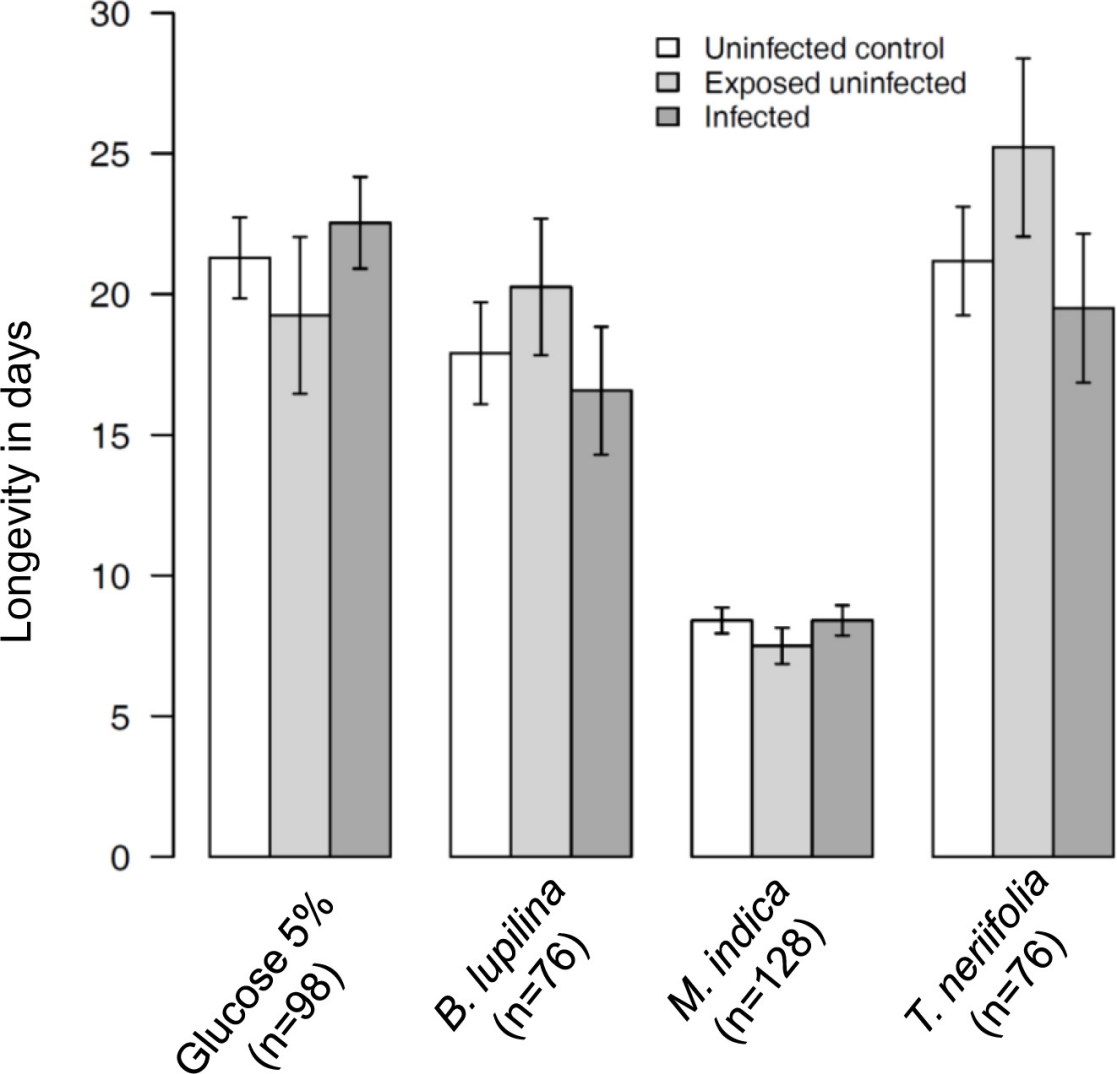
Effects of sugar treatment and infection status on mosquito longevity. Bars show the mean adult longevity ± 1 SE of uninfected control, exposed-uninfected and infected mosquitoes held on one of four sources of sugar. Numbers in brackets indicate, for each sugar treatment, the total number of mosquitoes monitored.

Infection status had no effect on mosquito lifespan (LRT *X* ^*2*^_2_ = 1; P = 0.6, Fig 3). Importantly, although mosquito longevity significantly varied among sugar treatment (LRT *X* ^*2*^_3_ = 160; P < 0.001, Fig 3), there was no sugar treatment by infection status interactions (LRT X^2^_6_ = 4.8; P = 0.56, Fig 3), demonstrating that the source of the sugar meals affected mosquito longevity irrespective of their infection status. We further analyzed the effect of infection status on the longevity of *T*. *neriifolia*-fed mosquitoes only and found no significant difference (LRT X^2^_2_ = 1.6; P = 0.4). This suggests that the observed reduction in the sporozoite index of mosquitoes fed on *T*. *neriifolia* does not result from the influence of increased parasite virulence on mosquito lifespan.

### Plant sugar source affects the transmission intensity of malaria

**Fig 4:**
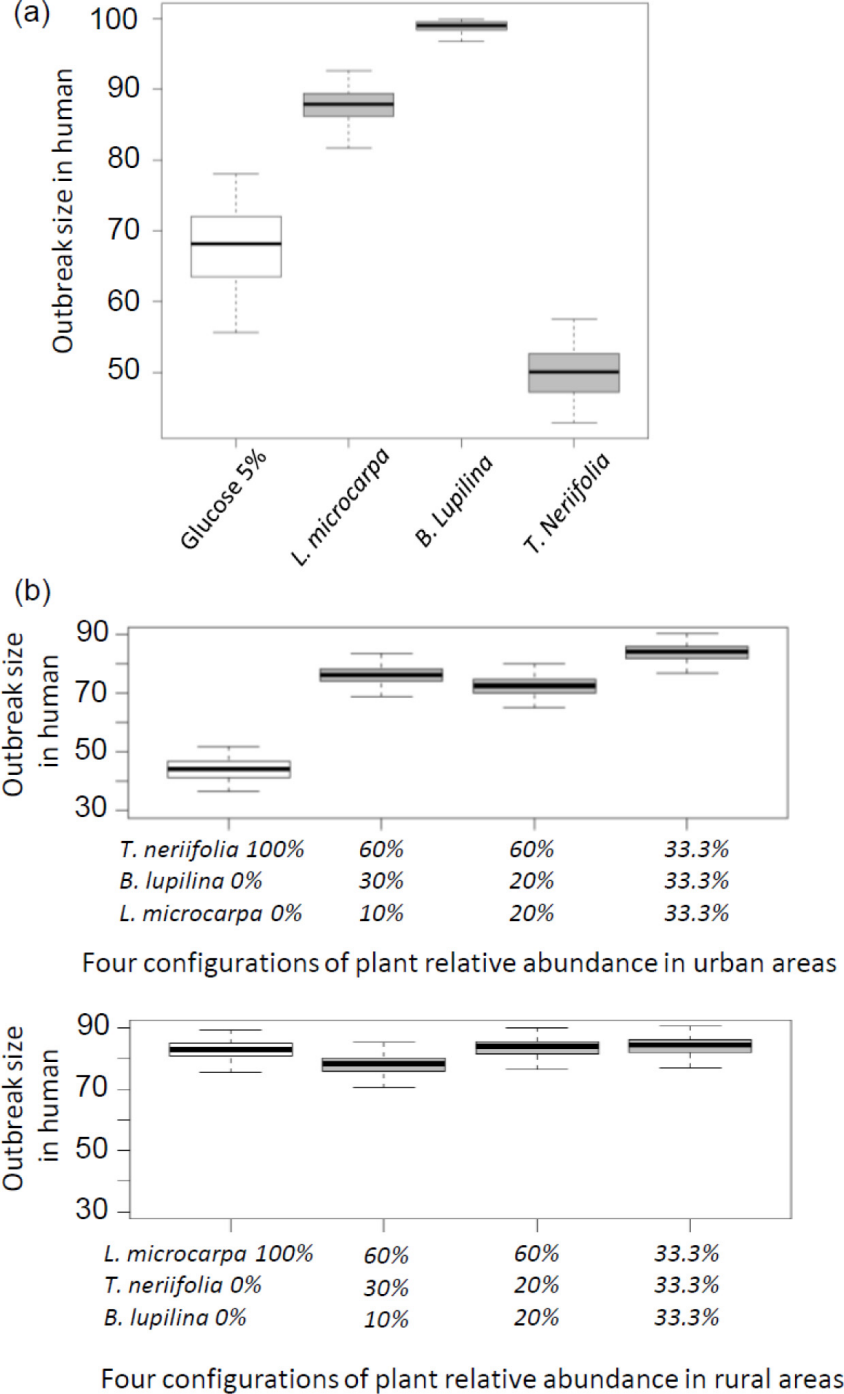
Theoretical difference in the distribution of outbreak sizes among plant sugars. (a) Epidemiological outcomes predicted by the model for each plant species considered in isolation, and (b) in a community perspective. In urban areas (upper panel), *Thevetia neriifolia* is relatively more abundant than *Barleria lupilina* and *Lannea microcarpa*. Four configurations with various relative abundances were considered: (i) a scenario of no diversity whereby *T*. *neriifolia*—the dominant plant—represents 100% of the feeding opportunities, (ii) a scenario of 60% *T*. *neriifolia*, 30% *B*. *lupilina* and 10% *L*. *microcarpa*, (iii) a scenario of 60–20–20%, and (iv) a scenario of equal feeding opportunities on each plant species. In rural areas (lower panel), *L*. *microcarpa* is relatively more abundant than *T*. *neriifolia* and *B*. *lupilina*. The same configurations as for urban areas were considered using this ranking. Outbreak size is the proportion of humans that has been infected at the end of the transmission season after one infectious human was introduced into the population. The box plots indicate the median (large horizontal bars), the 25^th^ and 75^th^ percentiles (squares), the minimum and maximum values (whiskers) and outliers (circles).

The epidemiological outcome predicted by the model for each plant species considered in isolation is consistent with intuitive expectations (Fig 4A). Compared to the baseline scenario with a 5% glucose solution, both *L*. *microcarpa* and *B*. *lupilina* caused an increased transmission potential, mainly because of increased infection rates among mosquitoes exposed to an infectious blood-meal (i.e. higher vector competence) (S4 Fig). In contrast, *T*. *neriifolia* induced a lower transmission potential, mainly because of a lower mosquito competence. as well as a longer infectious period (longevity).

When considering these individual effects within a community perspective, including the various proportions of each plant species, contrasting results appeared (Fig 4B). We assumed that mosquitoes do not display preference for plant species, hence that feeding choice is solely dependent on plant relative abundance. First, when considering plant communities generally occurring in urban areas, malaria transmission potential was largely impacted by plant relative abundance (Fig 4B). In this setting, increasing plant diversity leads to a decrease in *T*. *neriifolia* relative abundance and hence results in higher levels of transmission. In contrast, the different configurations of plant relative abundance showed similar patterns of transmission in rural areas where *T*. *neriifolia* is less abundant.

## Discussion

Feeding on different sources of natural plant-derived sugars can influence key traits that affect the capacity of mosquitoes to transmit malaria, including mosquito longevity and mosquito susceptibility to *P*. *falciparum*. When combined into an epidemiological model these effects have important consequences for malaria transmission. Compared to control mosquitoes fed on a 5% glucose solution, individuals fed on *T*. *neriifolia* showed a 30% decrease in malaria transmission. In contrast, mosquitoes fed on *L*. *microcarpa* and *B*. *lupilina* showed a 30% and 40% increase in transmission potential, respectively. Previous research has revealed the role of sugar in providing energy for flight, increasing mosquito survival and fecundity and decreasing biting rate on vertebrate hosts [12,23]. Our findings add a more direct effect of epidemiological importance by showing that plant-derived sugars can modulate mosquito-*Plasmodium* interactions.

The plant-mediated effect on infection could not be attributed to variation in blood meal size (S3 Appendix). Uptake of larger infectious blood meals may result in more gametocytes entering the mosquito midgut [41] and hence possibly increased infection. We tested whether plant sugar source could influence mosquito blood meal size. Mosquitoes fed with *L*. *microcarpa* prior to a blood meal took on average smaller blood meals than mosquitoes fed on glucose 5% or *B*. *lupilina* (S1 Appendix). However, infection in *L*. *microcarpa*-fed mosquitoes was not higher than that of glucose- or *B*. *lupilina*-fed individuals (Fig 2A). Furthermore, the results could not be attributed to mosquito inability to survive on particular plant sources such as *M*. *indica* owing to less feeding and eventual starvation (S4 Appendix).

At least three non-mutually exclusive mechanisms could explain the effects of plant sugar sources on mosquito competence for *P*. *falciparum*. First, there can be direct negative effects of plant secondary metabolites (e.g. alkaloids, terpenes, glycosides) on pathogen development. Secondary compounds generally play a role in plant defense against herbivores and thereby tend to be toxic and reduce host survivorship and reproduction [42,43]. These chemicals are found in all plant tissues, including floral nectars and fleshy fruits [44–47]. In bumblebees for example, the ingestion of alkaloids, terpenoids and iridoid glycosides contained in floral nectars can reduce infection by the protozoan parasite, *Crithidia bombi* [48,49]. Second, plants can enhance or reduce infection by modulating the host body condition and energetic status [50]. Feeding on poor quality resources may yield less energy to parasite growth and hence limit parasite infection [51]. Third, plants may indirectly influence infection through effects on host immune response or gut microbiota. Because immunological defenses are energetically expensive, hosts in poor condition may display reduced resistance to parasites [52]. In our experiments, mosquitoes fed with *M*. *indica* fruits or *T*. *neriifolia* flowers displayed reduced infection levels and reduced fitness (survivorship or fecundity), thus corroborating the first two mechanisms. *T*. *neriifolia* contain toxic cardiac glycosides, and mango fruits contain phenolic compounds, including mangiferin, two secondary metabolite groups suspected to have strong anti-protozoan properties [9,53–55]. In addition, previous studies have demonstrated that malaria parasites require sugars from the vector for an optimal development [56,57]. The relationships between nutrition, host general condition, toxic chemicals and infection are complex [58,59], and further studies are required to determine whether *T*. *neriifolia* and mango fruits have direct anti-plasmodium activities or have low nutritional value that limit both parasite growth and host fitness. The plant sugar source treatment was continuously provided in our study, thus it will be important to determine whether these effects result from the ingestion of plant-derived sugars prior (prophylactic effects) or post (therapeutic effects) infection. Mosquitoes from a colony continually replenished with F1 from wild-caught females were used here and it will also be important to study plant-mediated effects using field mosquitoes since rearing insects in the laboratory for many generations is unlikely to represent the genetic diversity observed in nature.

Under natural conditions, the frequency of contacts between a mosquito and a source of natural sugar will depend on the innate plant preference of the insect and environmental factors such as plant availability and accessibility. Our design did not allow the mosquitoes to choose between the different plants. We expect different epidemiological consequences when mosquitoes consume different proportions of each plant. Using our model, we simulated different scenarios of plant relative abundance in either urban (where *T*. *neriifolia* is relatively dominant compared to *B*. *lupilina* and *L*. *microcarpa*) or rural (where *L*. *microcarpa* dominates) areas. Our findings indicate that sharp epidemiological differences are indeed expected in urban areas where relatively small decreases in *T*. *neriifolia* abundance could lead to important increases in malaria transmission. This can be interpreted as an example of a dilution effect whereby poor-competent host species decreases the average efficiency of pathogen transmission [60,61]. In contrast, in rural areas, a decrease in *L*. *microcarpa* abundance in favor of *T*. *neriifolia* would only have a small negative impact on disease transmission (i.e. a weak dilution effect [60]).

Infected mosquitoes were more likely to carry eggs in their ovary compared to exposed-uninfected counterparts. This finding may illustrate a cost of resistance whereby the expression of an efficient immune response against *Plasmodium* establishment comes at a cost to egg production. Alternatively, this finding may illustrate the terminal investment hypothesis (also known as fecundity compensation), which postulates that when the future reproductive opportunities of an individual decline because of high mortality risk such as high infection levels, organisms increase their current reproductive investment [48]. We examined the first gonotrophic cycle only and further studies are needed to determine the mechanisms responsible for the increased probability of egg development in infected individuals.

Although we did not find any significant reductions in mosquito longevity or fecundity following *Plasmodium* infection, previous studies have demonstrated that this parasite can be costly to the mosquito host, especially in specific conditions reflecting what occurs in nature, such as nutritional, predation, insecticide or hydric stress [37,39,62–65]. Since *Plasmodium* infection decreases mosquito fitness, natural selection should favor the evolution of defenses against it. Besides immunological defenses, insect hosts can develop behavioral defenses including self-medication to better resist or tolerate their parasites [66,67]. Alternatively, malaria parasites might manipulate mosquito plant preference in ways that favor their own development and transmission [3,68]. A recent study found that *P*. *falciparum* infection increases both *An*. *gambiae* attraction to nectar sources and their total sugar uptake [69]. It will be of fundamental importance to better investigate the fitness costs and benefits of feeding on different natural sugar sources and to study plant preference by infected and uninfected mosquitoes. This will allow us to determine whether mosquito vectors or *P*. *falciparum* can exploit plant sugar sources to respectively self-medicate or manipulate the insect plant choice.

The interaction between malaria parasites and mosquito vector has drawn much attention for possible interventions to prevent transmission of this pathogen. Our findings have several implications for disease control. A number of different genetic manipulation strategies are now available to reduce mosquito competence for *Plasmodium* in the laboratory (refractory transgenic mosquitoes) [70–72]. In addition, the use of transmission-blocking vaccines against *P*. *falciparum* mosquito-stages has proven to be a promising strategy [73]. However, before translating these findings into the natural settings, it will be crucial to determine how the transgenes expression or vaccine efficacy can be affected by mosquito plant-feeding behavior. Finally, some novel perspective for malaria control including the cultivation of anti-*Plasmodium* plants may be truly sustainable. Concretely, fostering the planting of species such as *T*. *neriifolia*, which is commonly used by mosquitoes and which negatively affects vectorial capacity may locally reduce malaria transmission.

## Supporting Information

S1 Fig Natural plant species used in the experiments. (a) *Thevetia neriifolia* (syn: *Thevetia peruviana*. *Casacabela thevetia;* common name: yellow oleander. Fam: *Apocynaceae*) is an evergreen tropical shrub or small tree (up to 6 m) native of central South America. Yellow oleanders are common and widespread in villages and cities of West Africa where it is mostly used as a courtyard hedge. (b) *Barleria lupilina* (syn: *Barleria macrostachys;* common name: Hop-headed barleria; Fam: *Acanthaceae*) is a common ornamental shrub (about 1.5 m) in cities and villages of West Africa. (c) *Mangifera indica* (common name: mango; Fam: *Anacardiaceae*) is a large fruit-tree (up to 30 m) native of India, and widely distributed in West Africa for fruit consumption. In Burkina Faso, mango trees are commonly found in villages and cities within courtyards where they provide shade and fruit. (d) *Lannea microcarpa* (syn: *L*. *microcarpa acida*. *L*. *microcarpa djalonica;* common name: African grape. Fam: *Anacardiaceae*) is a tree (up to 15 m) indigenous of West Africa. Unlike mango, *L*. *microcarpa* species are not cultivated; they propagate naturally in the savanna vegetation and can occur in West African villages and cities.
(DOCX)

S2 Fig Sugar treatment layout. (a) Bundle of flowers of *Thevetia neriifolia*. (b) Bundle of flowers of *Barleria lupilina*. (c) Fruit of *Mangifera indica*. (d) Fruits of *Lannea microcarpa microcarpa*. (e) 5% glucose solution on cotton pads.
(DOCX)

S3 Fig Relationship between egg incidence and the number of oocysts in mosquito midgut. (DOCX)

S4 Fig Epidemiological outcome predicted by the model for each plant species considered in isolation when mosquito longevity (γ_i_) is similar across all sugar treatment. (DOCX)

S1 Appendix Field observational surveys, mosquito behavioural choice in the lab and Anthrone tests. (DOCX)

S2 Appendix Mathematical model details. (DOCX)

S3 Appendix Effects of plant sugar source on mosquito blood-meal size (protocol and results). (DOCX)

S4 Appendix Starvation experiments. (DOCX)

S1 Table Details of sample size, Infection rate and intensity in experiment 1. (DOCX)

S2 Table Details of sample size, Infection rate and intensity in experiment 2. (DOCX)

S3 Table Risk of mosquito mortality (hazard ratio) along with the 95%CI, z, P-value and sample size for each treatment group relative to the 5% glucose solution. (DOCX)
