# Supplementary material for: Plant-Mediated Effects on Mosquito Capacity to Transmit Human Malaria
Source: PLoS Pathog. 2016 Aug 4;12(8):e1005773. doi: 10.1371/journal.ppat.1005773 (PMC4973987; doi:10.1371/journal.ppat.1005773)

**S1 Figure. Natural plant species used in the experiments**. (a) *Thevetia neriifolia* (syn: *Thevetia peruviana. Casacabela thevetia;* common name: yellow oleander. Fam: *Apocynaceae*) is an evergreen tropical shrub or small tree (up to 6 m) native of central South America. Yellow oleanders are common and widespread in villages and cities of West Africa where it is mostly used as a courtyard hedge. (b) *Barleria lupilina* (syn: *Barleria macrostachys;* common name: Hop-headed barleria; Fam: *Acanthaceae*) is a common ornamental shrub (about 1.5 m) in cities and villages of West Africa. (c) *Mangifera indica* (common name: mango; Fam: *Anacardiaceae*) is a large fruit-tree (up to 30 m) native of India, and widely distributed in West Africa for fruit consumption. In Burkina Faso, mango trees are commonly found in villages and cities within courtyards where they provide shade and fruit. (d) *Lannea microcarpa microcarpa* (syn: *L. microcarpa acida. L. microcarpa djalonica;* common name: African grape. Fam: *Anacardiaceae*) is a tree (up to 15 m) indigenous of West Africa. Unlike Mango, *L. microcarpa* species are not cultivated; they propagate naturally in the savanna vegetation and can occur in West African villages and cities.


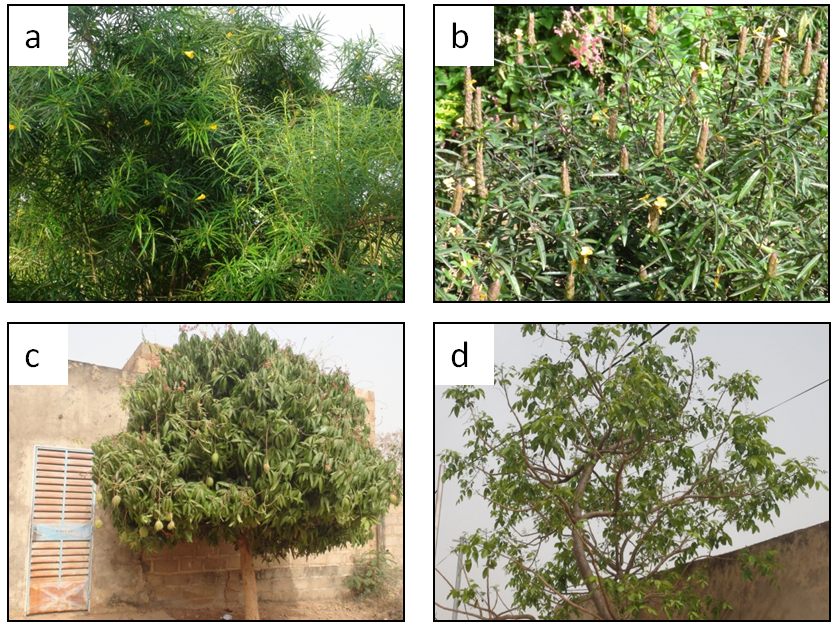

Supplement: S1 Fig — (a) Thevetia neriifolia (syn: Thevetia peruviana. Casacabela thevetia; common name: yellow oleander. Fam: Apocynaceae) is an evergreen tropical shrub or small tree (up to 6 m) native of central South America. Yellow oleanders are common and widespread in villages and cities of West Africa where it is mostly used as a courtyard hedge. (b) Barleria lupilina (syn: Barleria macrostachys; common name: Hop-headed barleria; Fam: Acanthaceae) is a common ornamental shrub (about 1.5 m) in cities and villages of West Africa. (c) Mangifera indica (common name: mango; Fam: Anacardiaceae) is a large fruit-tree (up to 30 m) native of India, and widely distributed in West Africa for fruit consumption. In Burkina Faso, mango trees are commonly found in villages and cities within courtyards where they provide shade and fruit. (d) Lannea microcarpa (syn: L. microcarpa acida. L. microcarpa djalonica; common name: African grape. Fam: Anacardiaceae) is a tree (up to 15 m) indigenous of West Africa. Unlike mango, L. microcarpa species are not cultivated; they propagate naturally in the savanna vegetation and can occur in West African villages and cities. (DOCX) [file ppat.1005773.s001.docx]
