# Supplementary material for: Plant-Mediated Effects on Mosquito Capacity to Transmit Human Malaria
Source: PLoS Pathog. 2016 Aug 4;12(8):e1005773. doi: 10.1371/journal.ppat.1005773 (PMC4973987; doi:10.1371/journal.ppat.1005773)
Supplement: S2 Fig — (a) Bundle of flowers of Thevetia neriifolia. (b) Bundle of flowers of Barleria lupilina. (c) Fruit of Mangifera indica. (d) Fruits of Lannea microcarpa microcarpa. (e) 5% glucose solution on cotton pads. (DOCX) [file ppat.1005773.s002.docx]

**S2
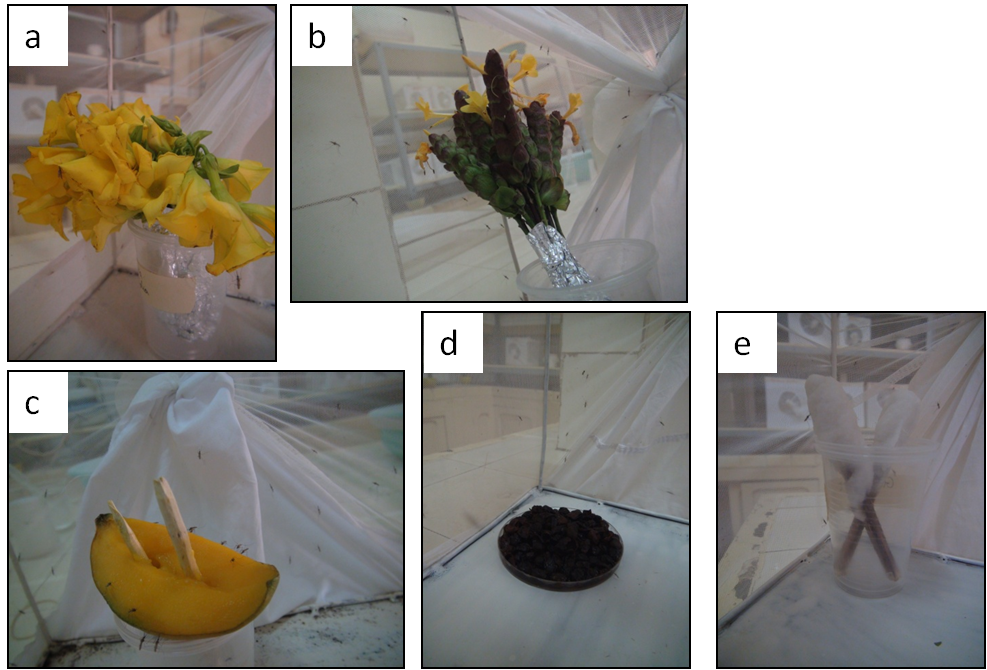
 Figure: Sugar treatment layout**. (a) Bundle of flowers of *Thevetia neriifolia.* (b) Bundle of flowers of *Barleria lupilina*. (c) Fruit of *Mangifera indica.* (d) Fruits of *Lannea. microcarpa microcarpa.* (e) 5% glucose solution on cotton pads.
