# Supplementary figures and images for: Plant-Mediated Effects on Mosquito Capacity to Transmit Human Malaria
Source: PLoS Pathog. 2016 Aug 4;12(8):e1005773. doi: 10.1371/journal.ppat.1005773 (PMC4973987; doi:10.1371/journal.ppat.1005773)

**
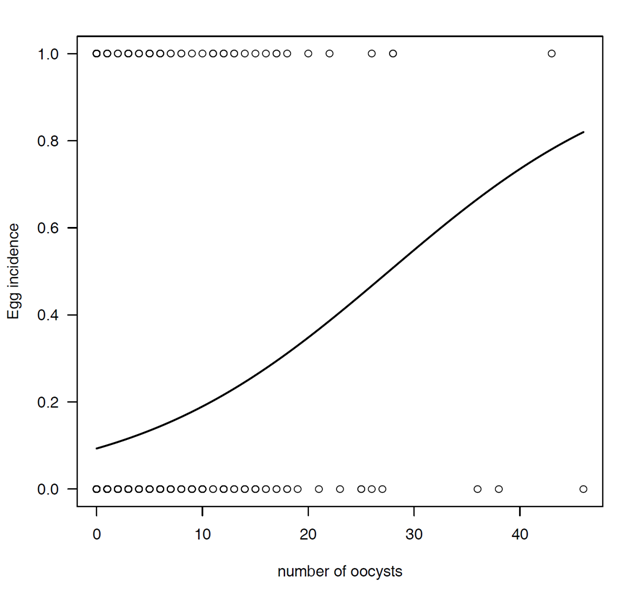
S3 Figure: Relationship between egg incidence and the number of oocysts in mosquito midgut.**

Supplement: S3 Fig — (DOCX) [file ppat.1005773.s003.docx]
