# Supplementary material for: Plant-Mediated Effects on Mosquito Capacity to Transmit Human Malaria
Source: PLoS Pathog. 2016 Aug 4;12(8):e1005773. doi: 10.1371/journal.ppat.1005773 (PMC4973987; doi:10.1371/journal.ppat.1005773)
Supplement: S4 Fig — (DOCX) [file ppat.1005773.s004.docx]

**Figure S4. Epidemiological outcome predicted by the model for each plant species considered in isolation when mosquito longevity (γ_i_) is similar across all sugar treatment**. To explore how much of the results in Fig. 4 (a) are due to plant effects on mosquito longevity vs on infection (i.e. examining the relative importance of longevity and infection effects on overall malaria transmission), the model was run by considering the sugar-mediated effect on mosquito infection only (everything else being equal). The differences in outbreak size are only due to differences in infection levels across sugar treatment.

L. microcarapa

Glucose 5%
