# Supplementary material for: Plant-Mediated Effects on Mosquito Capacity to Transmit Human Malaria
Source: PLoS Pathog. 2016 Aug 4;12(8):e1005773. doi: 10.1371/journal.ppat.1005773 (PMC4973987; doi:10.1371/journal.ppat.1005773)
Supplement: S1 Appendix — (DOCX) [file ppat.1005773.s005.docx]

**S1 appendix: Field observational surveys, mosquito behavioural choice in the lab and Anthrone tests**

**Field observational surveys**

This preliminary field survey was conducted to determine whether some of the plant species used here could serve as resting sites in nature. Using the same collecting technique as in Gouagna et al. 2010 [1] (i.e. clay pots suspended on plants amongst the most common found within peridomestic areas including *M. indica, T. neriifolia,* and *B. lupilina*), we captured female anopheles in the field (figure 1, a-b and table 1 below).


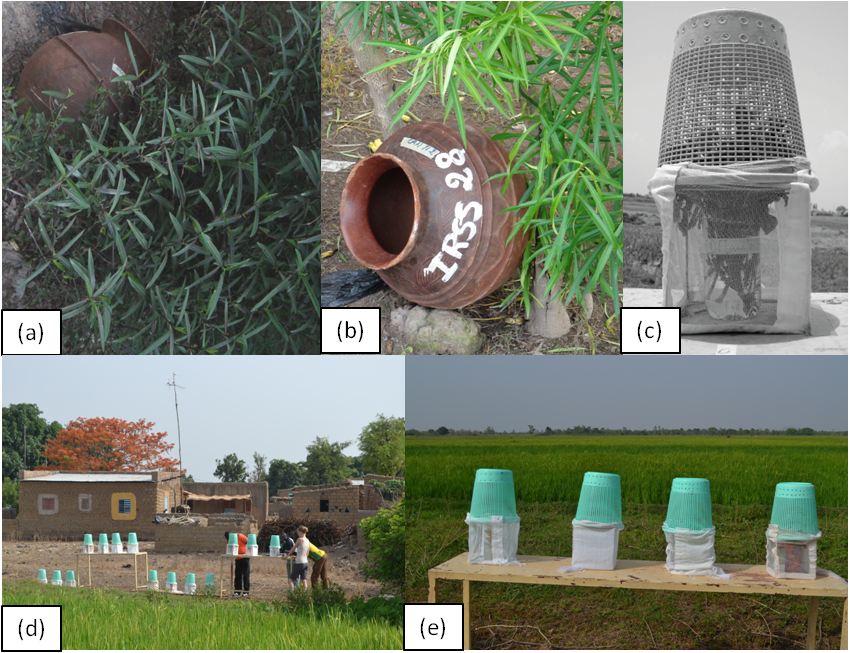


Figure 1. Claypots in wild *Barleria lupilina* (a) and *Thevetia neriifolia* (b)

| **plant species** | **no. of female *An. gambiae* *s.l.* captured** | **from n claypots** |
| --- | --- | --- |
| *Mangifera indica* | 7 | n=3 |
| *Barleria lupilina* | 6 | n=1 |
| *Thevetia neriifolia* | 15 | n=4 |

**Table 1. Number of *Anopheles* gambiae s.l. females collected during one night in three individual mango trees, one individual *Barleria lupilina* and four individual *Thevetia neriifolia* (one claypot per individual plant).**

**Behavioral assay**

The response of *An. coluzzii* females to (i) *Lannea microcarpa*, (ii) *Barleria lupilina*, (iii) *Thévetia neriifolia,* (iv) 5% glucose, and (v) water as control was studied in the laboratory using baited traps. The experiments were conducted with different batches of female mosquitoes (i) 5 days, (ii) 10 days and (iv) 17 days post-emergence. Mosquitoes were held in 30 × 30 × 30 cm cages with access to a 5 % glucose solution. In the afternoon of the experimental day, mosquitoes were retrieved from their holding cages and placed in paper cups with access to water only. At 6 pm, about 20 mosquitoes (range 13-25, see details in table 2 below) were released together overnight into one of five large 1m^3^ cage (Fig. 2A). Inside each large releasing cage, five 15cm^3^ odour-baited traps where positioned on the ground (Fig. 2B). These traps enclosed one of five sugar treatments: (i) *Lannea microcarpa*, (ii) *Barleria lupilina*, (iii) *Thévetia neriifolia* (Figure 2)*,* (iv) 5% glucose, or (v) water as control. The traps consisted of net which was raised 3 cm above the ground to allow mosquitoes to enter the traps. During the test, mosquitoes that were activated by the treatment stimuli flew through the 3 cm space and entered the trap. Although unlikely, using this device cannot completely exclude the possibility that mosquitoes that entered a given trap left that trap to either return into the releasing cage or fly into another trap. At 6 am at the end of the test, mosquitoes were either retrieved from the traps or from the releasing cage using a mouth aspirator and counted. The temperature in the experimental room was 27.5 ± 2.5 °C and the relative humidity 70 ± 10 %. The position of the traps was alternated between the large cages and between the testing nights to avoid any position effect. The *relative attractiveness* (proportion of mosquitoes caught in a given trap out of the total number retrieved from all traps) was gauged.

**
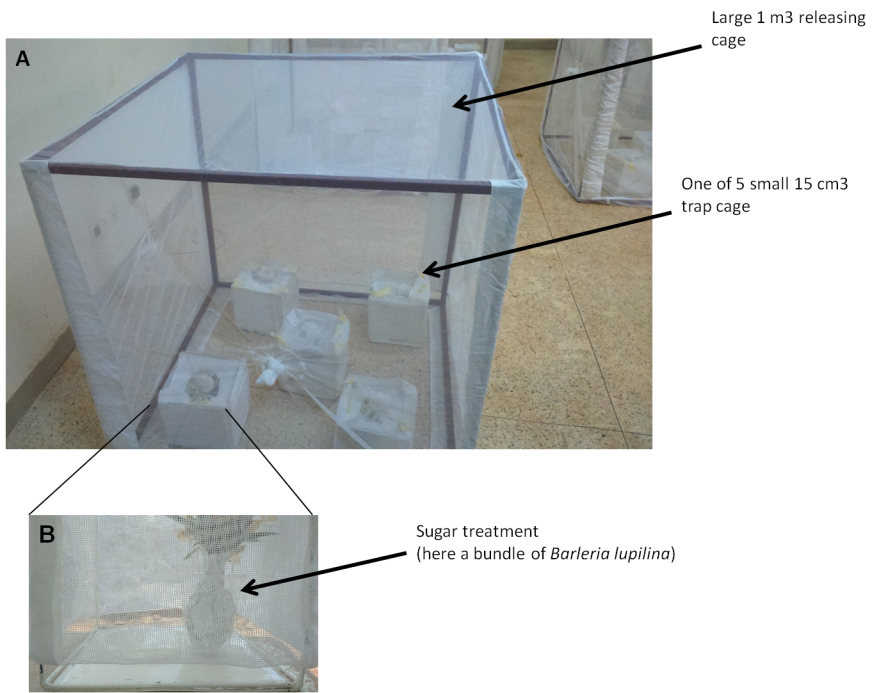
Figure 1. Behavioral device used for studying mosquito plant preference.** (A) A large 1 m3 mesh-covered cage into which mosquitoes were released. Each releasing cage contained 5 small 15 cm3 trap cages. (B) Bottom view of a small 15 cm3 trap cage. The net of the trap were rose by 3 cm to allow mosquitoes to enter the trap.

**Figure 3: Mean relative attractiveness of each sugar treatment to infected and uninfected mosquitoes at three periods.**

**Table 2. Results of the behavioral choice assay. These data were used to calculate the mean relative attractiveness shown in Figure 3.**

| **treatment** | **Mosquito Age** | **Replicates** | **No. of trapped mosquitoes in the treatment** | **Total number of trapped mosquitoes in all treatments** | **Relative attractiveness** | **Total number of released mosquitoes** |
| --- | --- | --- | --- | --- | --- | --- |
| Water | 5 | A | 1 | 9 | 0.111111111 | 20 |
|  |  | B | 0 | 6 | 0 | 20 |
|  |  | C | 0 | 8 | 0 | 20 |
|  |  | D | 1 | 7 | 0.142857143 | 19 |
|  |  | E | 0 | 4 | 0 | 20 |
|  | 10 | A | 2 | 7 | 0.285714286 | 25 |
|  |  | B | 1 | 4 | 0.25 | 22 |
|  |  | C | 1 | 3 | 0.333333333 | 13 |
|  |  | D | 0 | 6 | 0 | 24 |
|  |  | E | 0 | 8 | 0 | 19 |
|  | 17 | A | 1 | 4 | 0.25 | 21 |
|  |  | B | 9 | 15 | 0.6 | 26 |
|  |  | C | 0 | 9 | 0 | 22 |
|  |  | D | 2 | 12 | 0.166666667 | 25 |
|  |  | E | 0 | 15 | 0 | 24 |
| 5% glucose | 5 | A | 2 | 9 | 0.222222222 | 20 |
|  |  | B | 0 | 6 | 0 | 20 |
|  |  | C | 1 | 8 | 0.125 | 20 |
|  |  | D | 1 | 7 | 0.142857143 | 19 |
|  |  | E | 0 | 4 | 0 | 20 |
|  | 10 | A | 0 | 7 | 0 | 25 |
|  |  | B | 0 | 4 | 0 | 22 |
|  |  | C | 0 | 3 | 0 | 13 |
|  |  | D | 1 | 6 | 0.166666667 | 24 |
|  |  | E | 2 | 8 | 0.25 | 19 |
|  | 17 | A | 0 | 4 | 0 | 21 |
|  |  | B | 0 | 15 | 0 | 26 |
|  |  | C | 3 | 9 | 0.333333333 | 22 |
|  |  | D | 3 | 12 | 0.25 | 25 |
|  |  | E | 4 | 15 | 0.266666667 | 24 |
| *Lannea microcarpa* | 5 | A | 1 | 9 | 0.111111111 | 20 |
|  |  | B | 3 | 6 | 0.5 | 20 |
|  |  | C | 2 | 8 | 0.25 | 20 |
|  |  | D | 1 | 7 | 0.142857143 | 19 |
|  |  | E | 0 | 4 | 0 | 20 |
|  | 10 | A | 1 | 7 | 0.142857143 | 25 |
|  |  | B | 1 | 4 | 0.25 | 22 |
|  |  | C | 0 | 3 | 0 | 13 |
|  |  | D | 1 | 6 | 0.166666667 | 24 |
|  |  | E | 3 | 8 | 0.375 | 19 |
|  | 17 | A | 1 | 4 | 0.25 | 21 |
|  |  | B | 1 | 15 | 0.066666667 | 26 |
|  |  | C | 0 | 9 | 0 | 22 |
|  |  | D | 0 | 12 | 0 | 25 |
|  |  | E | 1 | 15 | 0.066666667 | 24 |
| *Barleria lupilina* | 5 | A | 3 | 9 | 0.333333333 | 20 |
|  |  | B | 3 | 6 | 0.5 | 20 |
|  |  | C | 5 | 8 | 0.625 | 20 |
|  |  | D | 2 | 7 | 0.285714286 | 19 |
|  |  | E | 1 | 4 | 0.25 | 20 |
|  | 10 | A | 2 | 7 | 0.285714286 | 25 |
|  |  | B | 0 | 4 | 0 | 22 |
|  |  | C | 2 | 3 | 0.666666667 | 13 |
|  |  | D | 4 | 6 | 0.666666667 | 24 |
|  |  | E | 3 | 8 | 0.375 | 19 |
|  | 17 | A | 0 | 4 | 0 | 21 |
|  |  | B | 3 | 15 | 0.2 | 26 |
|  |  | C | 4 | 9 | 0.444444444 | 22 |
|  |  | D | 3 | 12 | 0.25 | 25 |
|  |  | E | 4 | 15 | 0.266666667 | 24 |
| *Thevetia neriifolia* | 5 | A | 2 | 9 | 0.222222222 | 20 |
|  |  | B | 0 | 6 | 0 | 20 |
|  |  | C | 0 | 8 | 0 | 20 |
|  |  | D | 2 | 7 | 0.285714286 | 19 |
|  |  | E | 3 | 4 | 0.75 | 20 |
|  | 10 | A | 2 | 7 | 0.285714286 | 25 |
|  |  | B | 2 | 4 | 0.5 | 22 |
|  |  | C | 0 | 3 | 0 | 13 |
|  |  | D | 0 | 6 | 0 | 24 |
|  |  | E | 0 | 8 | 0 | 19 |
|  | 17 | A | 2 | 4 | 0.5 | 21 |
|  |  | B | 2 | 15 | 0.133333333 | 26 |
|  |  | C | 2 | 9 | 0.222222222 | 22 |
|  |  | D | 4 | 12 | 0.333333333 | 25 |
|  |  | E | 6 | 15 | 0.4 | 24 |

**Cold Anthrone.**

Cold anthrone test was performed to verify the presence of fructose in mosquitoes fed on each plant species [2]. When this chemical is revealed in mosquito gut/crop it means that the individual last fed on natural sources of plant sugar [3,4]. Twenty-five (25) mosquitoes from each treatment group were randomly selected for cold anthrone test to confirm their sugar ingestion. Anthrone solution was prepared by dissolving 150 mg of anthrone powder in 100 ml of 68,11% sulfuric acid buffer and kept in the fridge at 4°C. The anthrone solution is lemon yellow and acts specifically with fructose to give light green, blue or dark blue color depending on fructose quantity (Figure S3). Sugar sources were available all day and night long and the tests were performed at 11 am. Mosquitoes were individually crushed in different hemolytic tubes in 0.5 ml of prepared anthrone solution. The homogenates were incubated during 60 minutes at room temperature for color revelation with 3 tubes containing 0.5 ml of anthrone solution only as a negative control.

Of the 25 mosquitoes tested for each plant species, only 2 were negative for the anthrone test in the *B. lupilina* treatment, 0 for *L. microcarpa*, 3 for mango fruits, and 2 for *T. neriifolia* (see table and figure below). The proportion of positive mosquitoes for fructose was similar among the 4 natural sources of sugars (Pearson's Chi-squared test, *X^2^*=2.9, df=3, P=0.4).

| **Treatment** | **Total** | **Positive** | **Negative** | **Proportion positive** |
| --- | --- | --- | --- | --- |
| *Barleria lupilina* | 25 | 23 | 2 | 0.92 |
| *Lannea microcarpa* | 25 | 25 | 0 | 1.00 |
| *Mangifera indica* | 25 | 22 | 3 | 0.88 |
| *Thevetia neriifolia* | 25 | 23 | 2 | 0.92 |
| 5% glucose | 25 | 0 | 25 | 0.00 |
|  | | | | |

**Figure depicting the results of the cold anthrone tests**. Light green, blue or dark blue color indicates fructose-positive mosquitoes (i.e. mosquitoes that fed on the plant sugar source). Note that all glucose-fed mosquitoes are negative for the anthrone test (which acts specifically with fructose).

Literature cited

1. Gouagna L, Poueme RS, Dabiré KR, Ouédraogo J, Fontenille D, Simard F. Patterns of sugar feeding and host plant preferences in adult males of An . gambiae ( Diptera : Culicidae ). 2010;35: 267–276.

2. Van Handel E. The detection of nectar in mosquitoes. Mosq News. 1972;32: 458.

3. Gu WD, Muller G, Schlein Y, Novak RJ, Beier JC. Natural plant sugar sources of *Anopheles* mosquitoes strongly impact malaria transmission potential. PLoS One. 2011;6: e15996. doi:e15996 10.1371/journal.pone.0015996

4. Gary RE, Foster WA, Entomology V. *Anopheles gambiae* feeding and survival on honeydew and extra-floral nectar of peridomestic plants. Med Vet Entomol. 2004;18: 102–107. doi:10.1111/j.0269-283X.2004.00483.x
