# Supplementary material for: Plant-Mediated Effects on Mosquito Capacity to Transmit Human Malaria
Source: PLoS Pathog. 2016 Aug 4;12(8):e1005773. doi: 10.1371/journal.ppat.1005773 (PMC4973987; doi:10.1371/journal.ppat.1005773)
Supplement: S2 Appendix — (DOCX) [file ppat.1005773.s006.docx]

**S2 appendix: Mathematical model**

To extrapolate potential epidemiological outcomes of plant feeding habits, we used a simple model relying on the SIR framework [1,2] as following:

$$\frac{dS_{i}}{dt}=p_{i}\mu_{i}N_{i}-\left( abI_{H}+\mu_{i} \right)S_{i}$$

$$\frac{dE_{i}}{dt}=abI_{H}S_{i}-\left( \varepsilon_{i}+\mu_{i} \right)E_{i}$$

$$\frac{dI_{i}}{dt}=\varepsilon_{i}E_{i}-\left( \sigma_{i}+\mu_{i} \right)I_{i}$$

$$\frac{dU_{i}}{dt}=\sigma_{i}E_{i}-\gamma_{i}U_{i}$$

$$\frac{dS_{H}}{dt}=-acS_{H}\sum_{i=1}^{3} \tau_{i}I_{i}+\eta_{i}U_{i}$$

$$\frac{dI_{H}}{dt}=acS_{H}\sum_{i=1}^{3} \tau_{i}I_{i}+\eta_{i}U_{i}$$

where *S_i_* and *E_i_* represents abundance of susceptible and exposed (infected but not yet infectious) mosquitoes, respectively, who fed on plant species *i.* The model accounts for variation in vector competence and infectious potential (the period during which the mosquito can transmit the pathogen) over two phases: a first phase of low infection level (when the oocysts crack up and sporozoites begin to invade mosquito salivary glands, i.e. day 9 to days 10-11, see figure 2C) followed by a second phase of higher infection levels (from days 10-11 until mosquito death). Upon infection, exposed mosquitoes move to the class *I_i_*, which represents infectious mosquito during the first phase where a proportion τ_i_ are infectious. Finally, the mosquitoes move to the second infectious phase (*U_i_*) at rate $\sigma_{i}$ where η_i_ % are infectious and die at a rate $\gamma_{i}$. The parameters definitions and values are shown in the table below***.***

In the model, we quantify the number of humans who gets infected during a single season (i.e. the outbreak size) to estimate the influence of plant diversity on pathogen transmission. To derivate robust estimates, we apply a Latin Hypercube Sampling (LHS [3]) to explore the average behavior over possible values within standard deviation of different parameters changing with plant diversity. Simulations begun with one infectious individual and *S_i_* was set to the total vector abundance N times the proportion fixed by the parameter *p_i_*. After analyzing the individual impact of each plant species, we explored the role of plant community on epidemiological transmission. We assumed two different settings reflecting urban and rural areas, respectively. In urban areas of Burkina Faso, the relative abundance of the plant species used here is as follow: *T. neriifolia > B. lupilina > L. microcarpa*. In contrast, in rural areas of Burkina Faso, the relative abundance is *L. microcarpa* > *T. neriifolia > B. lupilina.*

For each setting, we explored four distinct scenarios of plant relative abundance. We first considered no diversity, i.e. where the dominant plant represents 100% of the feeding opportunities. The second and third configurations explored situations where the dominant plant represents 60% of these opportunities, but the second and the third plant represent 30% and 10% or 20% and 20% respectively. The last configuration considered evenly distributed feeding opportunities.

**Values of model parameters. Number between brackets indicates standard deviation).**

| **Parameter** | **Definition** | **Value** |
| --- | --- | --- |
| p_i_ | Proportion of mosquitoes getting a meal on plant species *i* | Variable |
| a | Mosquito biting rate | Variable |
| τ_i_ | Competence of mosquitoes during the first infectious phase for each plant species | glucose: 0.1 (0.093)  *L. microcarpa*: 0.3 (0.116)  *B. lupilina*: 0.15 (0.1107)  *T. neriifolia* 0.025 (0.0484) |
| η_i_ | Competence of mosquitoes during the second infectious phase for each plant species | glucose: 0.4697 (0.1204)  *L. microcarpa*: 0.6757 (0.0871)  *B. lupilina*: 0.7154 (0.0776)  *T. neriifolia* 0.4627 (0.0847) |
| ε_i_ | Duration of exposed phase (infected. but not infectious) for each plant species | 8 days |
| σ_i_ | Duration of the first infectious phase for each plant species | Glucose: 2 days  *L. microcarpa*: 3 days  *B. lupilina*: 2 days  T. neriifolia: 2 days |
| γ_i_ | Duration of the second infectious phase for each plant species | glucose: 4.54 days (1)  *L. microcarpa*: 4.97 days (1)  *B. lupilina*: 9.12 days (1)  *T. neriifolia*: 3.207 days (1) |

Literature cited

1. Keeling MJ, Rohani P (2008) Modeling infectious diseases in humans and animals. Princeton University Press. p.

2. Roux O, Vantaux A, Roche B, Yameogo B, Dabiré KR, et al. (2016) Evidence for carry-over effects of predator exposure on pathogen transmission potential. Proc R Soc Lond B Biol Sci In press.

3. Iman R, Helton J, Campbell J (1981) An approach to sensitivity analysis of computer models, Part 1. Introduction, input variable selection and preliminary variable assessment. Journal of Quality Technology 13: 174–183.
