# Supplementary material for: Plant-Mediated Effects on Mosquito Capacity to Transmit Human Malaria
Source: PLoS Pathog. 2016 Aug 4;12(8):e1005773. doi: 10.1371/journal.ppat.1005773 (PMC4973987; doi:10.1371/journal.ppat.1005773)
Supplement: S3 Appendix — (DOCX) [file ppat.1005773.s007.docx]

**S3 appendix: Effects of plant sugar source on mosquito blood-meal size**

**Protocol**

In mosquitoes, imbibed sugar is stored temporarily in the esophageal diverticula, mainly the large ventral diverticulum (crop) that extends into the abdomen. The sugar is transferred gradually to the midgut where absorption occurs [1]. It is generally assumed that there is a negative relationship between sugar feeding and blood-meal size ([2] and refs therein). In our infection experiments, although our mosquitoes were starved for 24 hours before blood meal and their crop were presumably empty, and only fully-bloodfed females were used, we cannot completely exclude the possibility that plant sugar sources influenced blood-meal size. Such effect could have consequences on infection since uptake of larger infectious blood meal can result in more parasites entering the mosquito midgut [3] and presumably increased infection. To test this hypothesis we measured the effects of plant sugar source on mosquito blood-meal size. The protocol for plant feeding was similar to that used in all experiments described here. Briefly, upon emergence, thirty adult females of *An. coluzzi* were randomly assigned to one of four sugar treatment: 5% glucose, *Lannea microcarpa*, *Barleria lupilina* and *Thevetia neriifolia*. Mosquitoes were maintained on their treatment for two days and starved for 24 hours before receiving a blood-meal blood using direct membrane feeding assays. Following the blood-meal, fully-fed females were kept individually in *Drosophila* tubes for four days to allow complete blood digestion. Blood meal size was measured indirectly as the mass of haematin excreted by individual mosquitoes, as described in [4]. Excreted material was dissolved from the plastic tubes in 1% lithiumcarbonate solution. The amount of hematin, a by-product of the decomposition of hemoglobin, was estimated by measuring absorbance of the mixture compared with a standard curve made with porcine serum hematin (Sigma–Aldrich).

**Result**

There was a significant effect of treatment on hematin concentration (ANOVA, F_3,85_=3.8, P=0.02). Numbers in brackets indicate the sample size for each plant sugar source. Different letters above the bars denote statistically significant differences based on Tukey HSD tests

**S2 Fig. Effect of plant sugar source on mosquito blood-meal size.**

**References**

1. Clements AN (2011) The biology of mosquitoes. CABI Publishing. p.

2. Mostowy WM, Foster W a (2004) Antagonistic effects of energy status on meal size and egg-batch size of Aedes aegypti (Diptera: Culicidae). Journal of vector ecology 29: 84–93.

3. Pichon G, Awono-Ambene HP, Robert V (2000) High heterogeneity in the number of Plasmodium falciparum gametocytes in the bloodmeal of mosquitoes fed on the same host. Parasitol 121 ( Pt 2: 115–120. doi:10.1017/S0031182099006277.

4. Briegel H (1980) Determintion of uric acid and hematin in a single sample of excreta from blood-fed insects. Experientia 36: 1428. doi:10.1007/bf01960142.
