# Supplementary material for: Plant-Mediated Effects on Mosquito Capacity to Transmit Human Malaria
Source: PLoS Pathog. 2016 Aug 4;12(8):e1005773. doi: 10.1371/journal.ppat.1005773 (PMC4973987; doi:10.1371/journal.ppat.1005773)
Supplement: S4 Appendix — (DOCX) [file ppat.1005773.s008.docx]

*S4 Appendix. Starvation experiments.*

Two additional experiments were performed to test whether mosquitoes are not simply starving when fed with mangoes. If they do, we would expect mosquito survival on mangoes to be similar to that of mosquitoes maintained on nothing or on water only. Upon emergence, batches of female *An. coluzzii* from the colony were randomly assigned to one of four treatments: a 5% glucose solution, Mango fruits, water only and nothing. Plant sugar sources were offered to mosquitoes in 30 cm × 30 cm × 30 cm mesh-covered cages and mosquito mortality was recorded twice a day (at 0800 and 1700) for 7 days. Treatment had a significant effect on mosquito mortality (*X^2^*_3_ $\chi_{1}^{2}$= 243, p < 2.2e-16, Figure below) with mosquitoes surviving best on glucose 5% followed by mango, water and nothing, all pair wise comparisons being different:

Estimate Std. Error z value Pr(>|z|)

mango - glucose5% == 0 2.5400 0.4616 5.502 1.37e-07 ***

nothing - glucose5% == 0 7.0300 0.6280 11.194 < 1e-07 ***

water - glucose5% == 0 4.6968 0.5395 8.706 < 1e-07 ***

nothing - mango == 0 4.4900 0.4690 9.573 < 1e-07 ***

water - mango == 0 2.1567 0.3420 6.307 < 1e-07 ***

water - nothing == 0 -2.3332 0.3562 -6.550 < 1e-07 ***

**
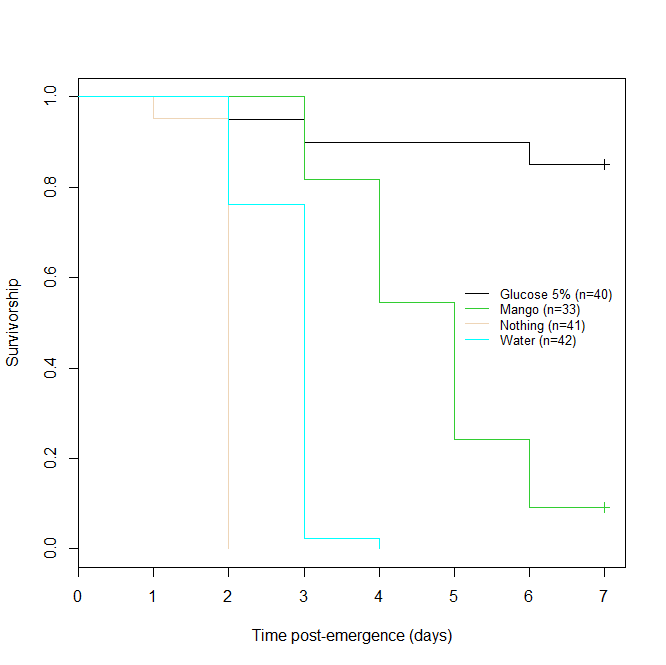
**

Second, we recorded the proportion of fully sugar-fed females maintained with either glucose 5% or mango fruits over a period of 4 days. 10 females were enclosed with 5% glucose and 10 with mango fruits. Twice a day (at 0800 and 1700) females were collected with a transparent mouth aspirator and the repletion status of each female was monitored. The results show that there was no statistically significant difference in the repletion rate of females exposed to a 5% glucose solution compared to mango fruits (Table below). Altogether, the results of these two experiments strongly suggest that mosquito females are not starving to death when maintained on mango fruits.

**Repletion score of female maintained with either a 5% glucose solution or a mango fruit.**

| days | period | 5% glucose repletion | *Mangifera indica* repletion | 5% glucose repletion rate (%) | *Mangifera indica* repletion rate (%) | Fisher’s exact test |
| --- | --- | --- | --- | --- | --- | --- |
| 1 | morning  evening | 0/10  2/10 | 0/10  0/10 | 0  0.2 | 0  0 | p=1  p=0.4 |
| 2 | morning  evening | 8/10  3/10 | 6/10  5/10 | 0.8  0.3 | 0.6  0.5 | p=0.6  p=0.65 |
| 3 | morning  evening | 5/10  0/10 | 4/10  3/8 | 0.5  0 | 0.4  0.375 | p=1  p=0.07 |
| 4 | morning | 5/10  4/10 | 2/7  1/3 | 0.5  0.4 | 0.286  0.333 | p=0.6  p=1 |
|  | evening |  |  |  |  |  |
| TOTAL |  | 27/80 | 21/68 | 0.34 | 0.31 | p=0.73 |

**
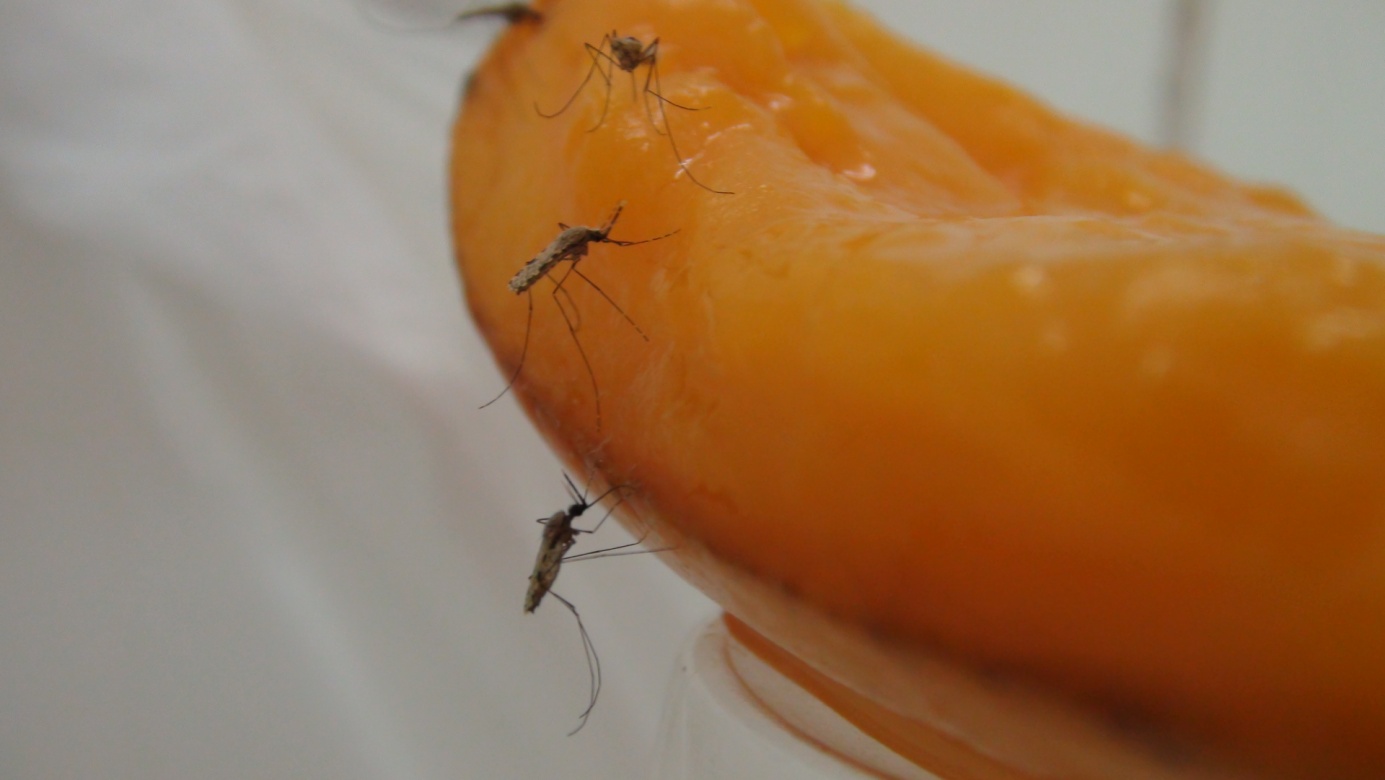
**

**Mosquito females of *An. coluzzi* feeding on mango fruits.** The female at the bottom of the picture presents sugar in its crop and midgut.
