# Supplementary material for: Plant-Mediated Effects on Mosquito Capacity to Transmit Human Malaria
Source: PLoS Pathog. 2016 Aug 4;12(8):e1005773. doi: 10.1371/journal.ppat.1005773 (PMC4973987; doi:10.1371/journal.ppat.1005773)
Supplement: S1 Table — (DOCX) [file ppat.1005773.s009.docx]

| **replicate** | **Gametocyte**  **carrier** | **Gametocyte**  **density** | **treatment** | **sample size** | **Infection**  **rate ± 95% CI** | **infection**  **intensity ± se** |
| --- | --- | --- | --- | --- | --- | --- |
| 1 | A | 168 | Glucose 5% | 50 | 0,5 ± 0,14 | 13,72 ± 2,48 |
|  |  |  | *B. lupilina* | 12 | 0,41 ± 0,27 | 13,4 ± 4,44 |
|  |  |  | *M. indica* | 1 | 1 ± 0 | 9 ± 0 |
|  |  |  | *T. neriifolia* | 34 | 0,5 ± 0,16 | 4,41 ± 0,93 |
|  | B | 56 | Glucose 5% | 44 | 0,23 ± 0,12 | 1,4 ± 0,4 |
|  |  |  | *B. lupilina* | 18 | 0,17 ± 0,17 | 1,33 ± 0,33 |
|  |  |  | *M. indica* | 4 | 0,25 ± 0,42 | 1 ± 0 |
|  |  |  | *T. neriifolia* | 31 | 0,19 ± 0,14 | 1,17 ± 0,16 |
| 2 | C | 88 | Glucose 5% | 35 | 0,43 ± 0,16 | 4,67 ± 1,49 |
|  |  |  | *B. lupilina* | 49 | 0,63 ± 0,13 | 4,61 ± 0,77 |
|  |  |  | *M. indica* | 50 | 0,42 ± 0,14 | 2,76 ± 0,5 |
|  |  |  | *T. neriifolia* | 50 | 0,32 ± 0,13 | 2,56 ± 0,32 |
| 3 | D | 48 | Glucose 5% | 53 | 0,28 ± 0,12 | 5,93 ± 1,31 |
|  |  |  | *B. lupilina* | 21 | 0,52 ± 0,21 | 3,18 ± 0,69 |
|  |  |  | *M. indica* | 24 | 0,04 ± 0,08 | 1 ± 0 |
|  |  |  | *T. neriifolia* | 50 | 0,44 ± 0,14 | 5,59 ± 1,31 |
|  | E | 16 | Glucose 5% | 17 | 0,35 ± 0,23 | 4,67 ± 2,1 |
|  |  |  | *B. lupilina* | 14 | 0,36 ± 0,25 | 6,6 ± 2,44 |
|  |  |  | *M. indica* | 11 | 0,09 ± 0,17 | 2 ± 0 |
|  |  |  | *T. neriifolia* | 32 | 0,56 ± 0,17 | 5,17 ± 0,76 |
| 4 | F | 104 | Glucose 5% | 17 | 0,82 ± 0,18 | 9,86 ± 2,14 |
|  |  |  | *B. lupilina* | 19 | 0,89 ± 0,14 | 9,88 ± 1,44 |
|  |  |  | *M. indica* | 11 | 0,45 ± 0,29 | 4,8 ± 1,59 |
|  |  |  | *T. neriifolia* | 16 | 0,63 ± 0,24 | 9,5 ± 2,18 |
|  | G | 64 | Glucose 5% | 43 | 0,49 ± 0,15 | 8,52 ± 2,15 |
|  |  |  | *B. lupilina* | 21 | 0,38 ± 0,21 | 6,25 ± 2,25 |
|  |  |  | *M. indica* | 10 | 0,4 ± 0,3 | 3 ± 0,81 |
|  |  |  | *T. neriifolia* | 27 | 0,55 ± 0,19 | 4,93 ± 0,88 |

**Table S1: Details of sample size, Infection rate and intensity in experiment 1**
