# Supplementary material for: Plant-Mediated Effects on Mosquito Capacity to Transmit Human Malaria
Source: PLoS Pathog. 2016 Aug 4;12(8):e1005773. doi: 10.1371/journal.ppat.1005773 (PMC4973987; doi:10.1371/journal.ppat.1005773)
Supplement: S2 Table — (DOCX) [file ppat.1005773.s010.docx]

| **replicate** | **Gametocyte**  **carrier** | **Gametocyte**  **density** | **treatment** | **sample size** | **Infection**  **rate ± 95% CI** |
| --- | --- | --- | --- | --- | --- |
| 1 | H | 168 | Glucose 5% | 13 | 0,64 ± 0,28 |
|  |  |  | *L. microcarpa* | 22 | 0,86 ± 0,14 |
|  |  |  | *B. lupilina* | 50 | 0,6 ± 0,13 |
|  |  |  | *T. neriifolia* | 6 | 0,25 ± 0,42 |
|  | I | 112 | Glucose 5% | 13 | 0,27 ± 0,26 |
|  |  |  | *L. microcarpa* | 38 | 0,47 ± 0,15 |
|  |  |  | *B. lupilina* | 50 | 0,64 ± 0,13 |
|  |  |  | *T. neriifolia* | 8 | 0,38 ± 0,33 |
| 2 | J | 152 | Glucose 5% | 0 | NA |
|  |  |  | *L. microcarpa* | 39 | 0,62 ± 0,15 |
|  |  |  | *B. lupilina* | 24 | 0,66 ± 0,18 |
|  |  |  | *T. neriifolia* | 27 | 0,48 ± 0,19 |
|  | K | 136 | Glucose 5% | 13 | 0,36 ± 0,28 |
|  |  |  | *L. microcarpa* | 32 | 0,56 ± 0,17 |
|  |  |  | *B. lupilina* | 46 | 0,63 ± 0,14 |
|  |  |  | *T. neriifolia* | 47 | 0,38 ± 0,14 |

**Table S2: Details of sample size and Infection rate in experiment 2**
