# Supplementary material for: Plant-Mediated Effects on Mosquito Capacity to Transmit Human Malaria
Source: PLoS Pathog. 2016 Aug 4;12(8):e1005773. doi: 10.1371/journal.ppat.1005773 (PMC4973987; doi:10.1371/journal.ppat.1005773)
Supplement: S3 Table — (DOCX) [file ppat.1005773.s011.docx]

| Experiment | Treatment | Hazard ratio (lower .95 - upper .95) | z | P-value | Sample size |
| --- | --- | --- | --- | --- | --- |
| 1 | Glucose |  |  |  | 238 |
|  | *B. lupilina* | 1.23 (0.67-2.27) | 0.67 | 0.502 | 172 |
|  | ***M. indica*** | **7.5 (4.8-11.7)** | **8.93** | **< 0.0001** | 221 |
|  | *T. neriifolia* | 1.16 (0.66-2.02) | 0.52 | 0.6 | 266 |
| 2 | Glucose |  |  |  | 271 |
|  | *L. microcarpa* | 0.8 (0.71-1.09) | -1.16 | 0.24 | 308 |
|  | ***B. lupilina*** | **0.6 (0.56-0.86)** | **-3.28** | **0.001** | 328 |
|  | *T. neriifolia* | 1.14 (0.92-1.41) | 1.15 | 0.25 | 253 |

**Table S3. Risk of mosquito mortality (hazard ratio) along with the 95%CI, z, P-value and sample size for each treatment group relative to the 5% glucose solution.**
